# A survey of perceptions and behavioural responses towards the COVID-19 pandemic in South Africa

**DOI:** 10.4102/phcfm.v17i1.4702

**Published:** 2025-01-17

**Authors:** Takana M. Silubonde, Catherine E. Draper, Shane A. Norris

**Affiliations:** 1 SAMRC Developmental Pathways for Health Research Unit, Faculty of Health Sciences, University of the Witwatersrand, Johannesburg, South Africa; 2 Global Health Research Institute, School of Human Development and Health, University of Southampton, Southampton, United Kingdom

**Keywords:** COVID-19, behaviour, perception, future pandemics, South Africa, survey

## Abstract

**Background:**

The South African response to the Severe acute respiratory syndrome coronavirus 2 (SARS-CoV-2) virus was swift and assertive, although it came with economic and social costs. An understanding of the pandemic experiences of different population groups is integral to enhancing disease control.

**Aim:**

The aim of this study was to identify behavioural responses and public perceptions relating to the coronavirus disease 2019 (COVID-19) pandemic.

**Setting:**

The study was conducted in South Africa.

**Methods:**

In June 2022, a national online survey (*N* = 3018) was conducted among adults (> 18 years). Logistic regression was used to examine the factors associated with adherence to government measures and vaccination. Structural equation modelling (SEM) was applied to examine the direct and indirect relationship of socioeconomic status (SES) with protective behaviours.

**Results:**

Data showed no direct association between SES and vaccination uptake, but SES was indirectly and positively associated with vaccination uptake. Socioeconomic status was also indirectly and positively associated with adherence to government measures through pathways mediated by access to the Internet, access to local news, government trust and positive government experiences.

**Conclusion:**

This study highlights the complexity of government measures and vaccination adoption and the socioeconomic barriers affecting these.

**Contribution:**

Results from this study should be used to inform future pandemic preparedness plans. In particular, policymakers should consider the importance of providing scientific information through channels accessible to each socioeconomic group to promote positive behavioural changes, as well as the need to adapt pandemic responses to different socioeconomic groups.

## Introduction

The coronavirus disease 2019 (COVID-19) pandemic significantly impacted the lives of individuals around the world. To contain the spread of the virus, governments called on citizens to adjust their social behaviours by putting into place preventative measures such as school and business closures, restrictions on movement, stay-at-home orders and restrictions on gatherings.^1^ Studies have shown that these preventative measures are effective in curbing the spread of disease.^2^ However, global perceptions of these preventative measures remain mixed. Fetzer et al. in their global study, reported that the majority of respondents had a positive perception of the measures put in place, and largely reported adhering to protective behaviours.^3^ Conversely, other studies have shown low-risk perceptions among some members of society.^4^ In particular, individuals with low health literacy, living in poverty and racial minority groups have been reported to have a low perception of risk posed by the COVID-19 pandemic, resulting in lower uptake of preventative behaviours among these groups.^5^ Furthermore, the pandemic is reported to have had significant negative economic, social and psychological impacts on the lives of individuals.^6,7^

In South Africa, the response to the Severe acute respiratory syndrome coronavirus 2 (SARS-CoV-2) virus was swift, effective and assertive, with the government implementing stringent lockdowns, with large economic costs.^8^ South Africa is a country with high levels of inequality^9^; hence, it is not surprising that the economic and social ramifications of the pandemic are reported to have disproportionately affected those already socioeconomically disadvantaged.^8^ Nevertheless, reports have highlighted how the once ‘cushioned’ middle class faced economic instability, along with the psychological stress experienced by higher income groups as a result of the pandemic.^10^

An understanding of the experiences of different population groups with the pandemic and how they perceive government responses to COVID-19, both generally and on specific responsibilities, is essential for identifying potential obstacles to achieving disease control objectives. As such, there have been numerous survey studies conducted in South Africa about the COVID-19 pandemic. These include the National Income Dynamics Survey – Coronavirus Rapid Mobile Survey (NIDS-CRAM, https://cramsurvey.org) to better understand the socioeconomic impact of the pandemic as well as the complexity of vaccine distrust and vaccine hesitancy.^11^ Other survey studies have reported on people’s willingness to have the vaccine; sources for COVID-19 information and communication about COVID-19; and factors associated with vaccine hesitancy, such as mistrust in government and job insecurity.^12,13^ While these studies^12,13^ have highlighted the complexity of South African adults’ willingness to have the COVID-19 vaccine, they focussed on vaccines and did not investigate other implemented government measures, or the impacts of the COVID-19 pandemic, as covered by the NIDS-CRAM.^11^ Further research to investigate all of these issues in the same sample of adults across South Africa was seen to be necessary to better understand the complexity of these issues.

Results from a qualitative study at the peak of the Omicron wave (December 2021) in South Africa provided more in-depth insights.^10^ This study highlighted factors that may have impeded the implementation of government measures, namely government mistrust, a lack of scientific understanding and poor communication. In addition, it was found that all income groups experienced psychological challenges, but economic impacts were experienced differently. For instance, those in lower-income groups spoke about an increase in financial strain, adding to their existing economic challenges. Middle-income earners reported experiencing increased job insecurity, whereas high-income earners did not report economic challenges.^10^ Although the focus groups provided insight into possible challenges and solutions to the pandemic across income groups, further work was needed to determine the extent to which these findings extended to a wider population in South Africa. Moreover, it is probable that public perception of the pandemic has changed throughout the pandemic. Hence, conducting a national survey, guided by the results of the qualitative study and drawing a sample from a range of income groups, provides an opportunity to verify our qualitative results as well as capture public perceptions at a later stage of the pandemic. This well-rounded understanding of public perceptions may be of benefit in planning for predicted future pandemics and other global challenges that the world is facing such as climate change.

Therefore, the aim of this study was to identify behavioural responses and public perceptions relating to the COVID-19 pandemic, specifically perceptions of technology and media, sources of information, trust in national and international bodies, COVID-19 restrictions, COVID-19 vaccination, and overall wellbeing and experience of COVID-19.

## Methods

### Study design

A national online survey was conducted in June 2022.

### Setting

The study setting was South Africa. South Africa is an upper-middle-income country, characterised by a range of urban and rural settings, multiple languages (12 official), as well as a diversity of cultures and ethnicities. The country still has vast inequities between economic groups, because of the legacies of *apartheid* and colonialism, and common socioeconomic challenges experienced include high rates of unemployment, poverty and food insecurity.

### Study population and sampling strategy

To access a diverse sample of participants for the survey we partnered with MoyaResearch (https://moya.app/research-panel/) with a national database of potential participants (~135 000 individuals, recruited from the larger MoyaApp audience of 6.5 million monthly active users). MoyaApp is a ‘data free’ super-app based in South Africa, which has amassed a large audience mainly because of the fact that the majority of services on the application do not require the user to have a positive mobile data balance to engage with them. Furthermore, even if a user has a positive mobile data balance, the app does not consume the user’s mobile data, but rather MoyaApp covers the data costs – similar to the concept of ‘toll-free’ calling. Because of the data-free nature of MoyaApp, the barrier to engaging with the digitally hard-to-reach (e.g. in low-income settings) has been lowered, thus, allowing researchers the opportunity to collect data from users who would normally experience mobile data issues when engaging with an electronic survey. The database of MoyaApp includes adults across all age groups, from all nine provinces in South Africa (urban and rural areas), covering a range of education levels, ethnicities, socioeconomic status and economic status.

Participants were contacted through the usual contacting procedures on the MoyaApp. Proportional quota sampling was used to ensure that respondents were demographically representative of the South African public, with quotas based on age, gender, province and socioeconomic status. Respondents were required to be 18 years or older and to speak English. Socioeconomic status was determined by the Living Standards Measure (LSM),^14^ which is a segmentation tool that is widely used in South Africa. It comprises 10 groups, with 10 being the highest living standard level and one the lowest. Given the online nature of the survey, it was not feasible to include LSM 1–4 (lowest living standard groups) given the challenges of network connectivity and access to a smartphone or computer that would be required to participate in an online survey. Given the virtual panel of participants recruited across South Africa, we aimed to have a minimum of 3000 participants complete the survey to ensure greater participant representativeness of the virtual panel. Our assumptions were that all eligible panel members were invited to participate and that participants who completed the survey would be at random. Also, a minimum of 3000 sample size would be sufficient to draw statistically significant conclusions with a high degree of confidence from a general population. We did not perform a power calculation.

### Data collection

Information about the survey was sent out electronically by MoyaResearch to all potential participants in their national database. After reading the respondent information, consent was implied if the person completed the survey and submitted it via the MoyaResearch website. A total of 3018 participants ultimately participated in the survey. Anonymised data were stored on the MoyaResearch platform and shared with the research team.

The survey questions covered participants’ behavioural responses and public perceptions relating to the COVID-19 pandemic, specifically perceptions of technology and media, sources of information, trust in national and international bodies, COVID-19 restrictions, COVID-19 vaccination and overall wellbeing and experience of COVID-19. Details of the survey are outlined in the Online Appendix Table 1-A1.

### Data analysis

Continuous data were tested for normality by visual inspection of Q-Q plots and histograms, and the Shapiro-Wilk test. Normally distributed data are expressed as means ± standard deviation (s.d.); non-normally distributed data are expressed as medians (interquartile range [IQR]). Discrete data were represented with the number count and percentage (*N*%). Multivariable logistic regression was used to examine the factors associated with adherence to government measures and vaccination. Models were constructed with the use of block stepwise regression whereby variables were entered into the model in blocks in order of anticipated importance using the authors’ judgement. None of the included variables showed multicollinearity, with variance inflation factors < 2 for each model.

**FIGURE 1:**
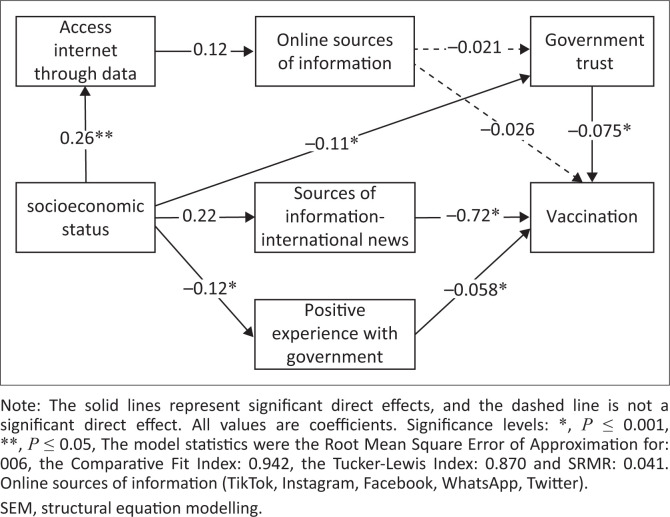
SEM framework for vaccination outcome.

**FIGURE 2:**
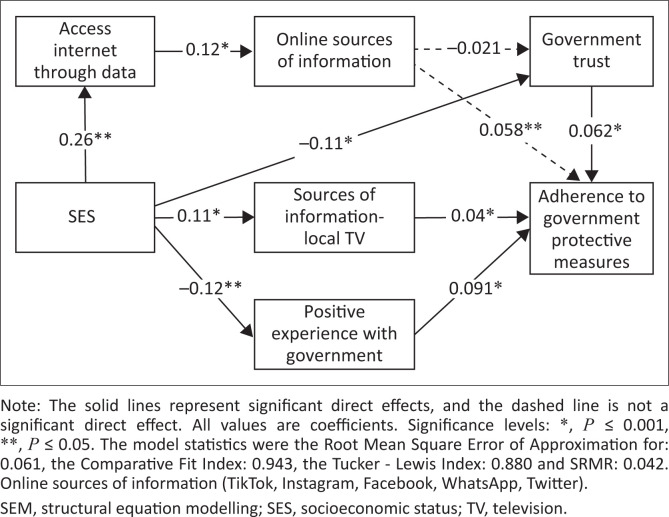
SEM framework for adherence to government protective measures outcome.

Structural equation modelling (SEM) was applied to examine the specific causal models and assess the comparative strength of direct and indirect relationships among independent variables with vaccination and adherence to government measures. Structural equation modelling was the analysis of choice as it allows for a pictographic representation of hypothesis-driven relationships between variables such as potential mediators, confounders and latent variables.^15^ The multivariable analyses were guided by an *a priori* model (Figure 1 and Figure 2), based on expert knowledge and literature. Bold lines represent statistically significant paths while dotted lines represent paths that were not statistically significant. We then hypothesised relationships *a priori* among the variables. From this framework, SEM was used to estimate the associations in the different pathways between access to devices (Wi-Fi and data), access to information (online and television sources), government trust, government experience, perception of government protective measures, perception of vaccination socioeconomic status factors and the two outcomes vaccination and adherence to government measures. Direct, indirect and total effects were calculated using nonlinear combination estimates.

To evaluate the best fitting model for our data, we reported goodness of fit indices including root mean squared error of approximation (RMSEA), comparative fit index (CFI), Tucker–Lewis index (TLI) and standardised root mean squared residual (SRMR). All data analyses were performed using Stata statistical software, version 14.1 (StataCorp, College Station, TX: Stata Corporation).

### Ethical considerations

Ethical approval for the study was obtained from the University of the Witwatersrand Human Research Ethics Committee (Non-Medical, reference no.: H21/10/06). Anonymised data were kept confidential on the secure MoyaResearch system.

## Results

### Characteristics of participants

The characteristics of participants in the study sample are presented in Table 1. Over the study time period, 3018 participants completed the survey. The median age of the participants was 32 years (IQR: 26–39), with 54.9% of participants identifying as female. The majority of participants were middle-income earners represented by a median LSM of 6 (IQR: 6–8). The highest level of education for most participants was a school leaving certificate (51.0%), with 13.2% having lower than secondary school education and 35.7% having a post-secondary school qualification. A total of 53.2% of participants were unemployed and 10.5% of participants were students. To access media 99.2% of participants used a smartphone, with 89.7% of participants accessing the internet using mobile data. The main sources of information were local news (75.2%), followed by social media (71.2%). More than half of the participants reported vaccination (62.8%) and 67.0 % of participants reported adhering to government measures (e.g. wearing a mask and physical distancing). Half the participants (50.3%) reported trust in the government, and the majority of participants (64.3%) reported positive previous experiences with the government.

**TABLE 1:** Characteristics of survey participants (*N* = 3018).

Variable	*n*	%
**Age (years)**		
18–34	1720	57.0
35–54	1195	39.7
55+	101	3.3
**Sex**		
Male	1360	45.1
Female	1658	54.9
**Education**		
No education	67	2.2
Some primary	37	1.2
Primary school	62	2.1
Secondary	232	7.7
Secondary school (school leaving certificate)	1539	51.0
College training (certificate, diploma)	810	26.8
Degree	163	5.4
Postgrad professional	98	3.2
Masters/doctorate	10	0.3
**LSM**		
5	744	24.7
6	1029	34.1
7	400	13.3
8	444	14.7
9	290	9.6
10	111	3.7
**Occupational status**		
Student	318	10.5
Employed	-	-
Part-time	336	11.1
Full-time	358	11.9
Self-employed	400	13.3
Unemployed	1606	53.2
**Devices used to access media**		
Smartphone	2995	99.2
Laptop at home	711	23.6
Tablet at home	273	9.0
Desktop at work	124	4.1
**Internet Access**		
Data on my phone	2707	89.7
Wi-Fi at home	545	18.1
Wi-Fi at work	308	10.2
Wi-Fi at public places	726	24.1
**Main sources of information**		
TV (local)	2271	75.2
TV (international)	826	27.4
Social media (TikTok, Instagram, Facebook WhatsApp, Twitter)	2159	71.5
**Vaccination**		
Yes	1896	62.8
**Adherence to government measures**		
Yes	2022	67.0
**Government trust**		
Yes	1518	50.3
**Positive government experience**		
Yes	1942	64.3

Note: Age (years): Median = 32, IQR = 26.0–39.0; LSM: Median = 6, IQR = 6.0–8.0.

IQR, interquartile range; LSM, living standards measure; TV, television.

Participants’ perceptions towards vaccination and future pandemics are presented in Table 2. When asked what should be done differently in the next pandemic, more than half of the participants (59.0%) said better government communication, 42.2% said less corruption and 36.2% reported that mandatory vaccination should be implemented. The majority of participants (68.3%) indicated that they were either unsure about booster shots or would not get them. Child vaccination acceptance was high with 68.3% of parents indicating that they would vaccinate their children if the vaccine was made available to them.

**TABLE 2:** Perceptions towards vaccination and future pandemics.

Question	*n*	%
**What should be done differently in future pandemics?**		
Better communication (yes)	1781	59.0
Less corruption (yes)	1285	42.6
Compulsory vaccination (yes)	1093	36.2
**What do you think about to have regular COVID-19 booster shots (e.g. every 6 months or 12 months)?**		
I will have mine as soon as possible	1239	41.0
I am still not sure if I will have one	1068	35.4
I do not want any booster shots	711	23.6
**If you have a child, would you have your child vaccinated against COVID-19?**		
Yes	1583	68.3
No	732	31.6

COVID-19, coronavirus disease 2019.

### Multivariable logistic regression

Table 3 depicts the results of a multivariable logistic regression model for factors associated with adherence to government measures and vaccination. Those who reported government trust were 0.5 times less likely to vaccinate (odds ratio [OR]: 0.56 [confidence interval {CI}: 0.47, 0.67] *p* < 0.001). Likewise, those who reported positive government experiences (OR: 0.67 [CI: {0.56, 0.80}] *p* < 0.001) and international news (OR: 0.76 [CI: 0.64, 0.9] *p* = 0.002) were less likely to vaccinate. Those who reported government trust (OR: 1.84 [CI: 1.53, 2.23] *p* < 0.001) and positive government experience (OR: 1.83 [1.53, 2.18] *p* < 0.001) were almost twice as likely to adhere to government measures. Those who used social media (OR: 1.26 [1.07, 1.5] *p* = 0.007) and local news (OR: 1.20 [1.01, 1.44] *p* = 0.043) as a source of information were more likely to adhere to government measures.

**TABLE 3:** Multivariable logistic model of factors associated with vaccination and adherence to government protective measures.

Variable	OR	95% CI	*p*
**Vaccination outcome**			
Positive experience with government	0.67	0.56; 0.80	< 0.001*
Government trust	0.56	0.47; 0.67	< 0.001*
International news	0.76	0.64; 0.91	0.002*
Local news	0.89	0.74; 1.10	0.170
**Adherence to government protective measures outcome**			
Social media	1.26	1.07; 1.50	0.007*
Government trust	1.84	1.53; 2.23	< 0.001*
Positive experience with the government	1.83	1.53; 2.18	< 0.001*
Local news	1.20	1.01; 1.44	0.043*

Note: Vaccination or adherence to protective behaviours (yes or no) was used as the outcome variable. Other variables were removed from the model because of a lack of significance.

CI, confidence interval; OR, odds ratio.

* , significant results.

### Structural equation modelling: Vaccination outcome

Results from the SEM analyses regarding the association between socioeconomic status (SES), access to devices, access to information, government trust, government experience and the outcome of vaccination are shown in Online Appendix Table 2-A1 and Figure 1. There was no direct association between SES and the outcome of vaccination uptake, but SES was indirectly and positively associated with vaccination uptake ([0.021] *p* ≤ 0.001) through pathways mediated by positive government experiences, access to international news and government trust. Positive, direct associations were observed between SES and the following: access to the Internet ([0.28] *p* ≤ 0.001), access to online sources ([0.037] *p* ≤ 0.001) and accessing international news ([0.22] *p* ≤ 0.001). Socioeconomic status was negatively associated with positive government experiences ([0.12] *p* ≤ 0.001) and government trust ([0.12] *p* ≤ 0.001). Government experiences ([−0.052] *p* ≤ 0.001), international news ([−0.72] *p* ≤ 0.001), trust in government ([−0.075] *p* ≤ 0.001) and government experiences ([−0.11] *p* ≤ 0.001) were directly and negatively associated with vaccination. Government trust and positive government experience were strongly associated ([0.69] *p* ≤ 0.001).

### Structural equation modelling adherence to government measures outcome

Results from the SEM analyses for the association between SES, access to devices, access to information, government trust, government experience and the outcome adherence to government measures are shown in Online Appendix Table 3-A1 and Figure 2. Positive government experience was positively directly ([0.091] *p* ≤ 0.001) and indirectly ([0.043] *p* ≤ 0.001) associated with adherence to government measures, with a total association of ([0.13] *p* ≤ 0.001). Government trust was positively and directly associated with adherence to government measures ([0.062] *p* ≤ 0.001), while SES was indirectly and positively associated with adherence to government measures ([0.023] *p* ≤ 0.001). Local news was positively and directly associated with adherence to government measures ([0.04] *p* ≤ 0.05).

## Discussion

This study reports on the perceptions and behaviour responses (e.g. adhering to restrictions and vaccination uptake) of the South African adult population at a later stage in the pandemic, as the South African government began to ease restrictions. Government trust, positive previous government experiences and sources of information increased the likelihood of adhering to government measures. However, in contrast, government trust, positive government experiences and sources of information did not appear to positively influence self-reported vaccination but instead were associated with reduced likelihood of vaccination. A surprising finding, given that adherence to government measures and vaccination, are both preventative measures; hence, it is probable that the behaviour surrounding them would be similar; however, this is not the case. A study in Kenya found similar results where adherence to government measures was linked to reduced willingness to vaccinate.^16^ It is possible to deduce from this that some individuals may have a preference towards government measures, possibly leading to reluctance to vaccination. However, this may need to be further investigated. Nonetheless, our findings draw attention to the complexity of vaccine hesitancy, which has been identified in previous South African COVID-19 studies,^11,12,13^ is not a new phenomenon in South Africa. In 2009, a child vaccination study identified vaccine hesitancy as one of the main challenges facing vaccination programmes,^17^ and a measles outbreak in South Africa between 2003 and 2011 was also linked to vaccine hesitancy.^18,19^

The results of the present study highlighted the positive association between socioeconomic status and vaccination uptake. South Africa is a highly inequitable country with a history of socioeconomic-related health inequality;^20,21^ as such, it is likely that previous existing factors linked to socioeconomic status may have influenced vaccination uptake. For example, lower education may lead to vulnerability to myths and misinformation, lower income may result in greater perceived barriers to obtaining COVID-19 vaccines, a geographical disadvantage may be linked to fewer opportunities to visit healthcare providers where vaccines can be recommended and higher experiences of discrimination linked to greater medical mistrust.^22,23,24,25^ Furthermore, studies in South Africa have reported a lower risk perception among lower income groups and how COVID-19 has been viewed as ‘a disease of privilege’.^10,26^ Hence, it is probable that if those in lower income groups perceive the possibility of getting the disease as unlikely and possibly perceive the vaccine and its side effects as dangerous, they are unlikely to vaccinate.^27^

Similarly, the results also showed that adherence to government protective measures such as social distancing and mask-wearing were indirectly and positively associated with socioeconomic status. This is in agreement with another South African study carried out by Swart et al. who reported higher adherence to protective measures among high-income earners.^28^ It is also important to note that our results highlighted how socioeconomic status strongly influenced access to information via both television and the Internet. In turn, access to information (local news and online sources) was positively associated with adherence to government protective measures. It is well evidenced that information sufficiency is an integral tool in increasing adherence and uptake of protective behaviours.^29^ It has been evidenced that population risk perceptions and individual behavioural responses to a pandemic can be improved upon by both the quantity and quality of information provided.^30,31^ However, if inequity is a barrier to obtaining information, the majority (56%) of South African citizens living below the poverty line may not receive important health-related information.^32^ As such, during times of health crisis, government communication must not only be clear and contain scientific information but the information must be accessible to the majority. The effects of lack of information are potentially detrimental in South Africa, as lack of information has been linked to vaccine hesitancy.^10,33^ Furthermore, information sufficiency could help address the hesitancy related to the vaccine booster as reported in this study.

Although some studies have reported on the harmful effects of misinformation being propagated on social media,^34,35,36,37^ our survey did not show that information obtained through these platforms decreased adherence to government protective measures and vaccination uptake. This may well reflect a bias in the sample of participants who responded to the survey, but this might also indicate that these platforms may influence individuals positively and negatively. Nevertheless, despite this study linking online platforms to increased protective behaviours, many participants indicated that they did not trust social media as a source of information.

As reported by other studies, government trust was reported to influence adherence to government protective measures.^38,39,40,41^ It is therefore not surprising that participants indicated that in future pandemics, there should be less government corruption and better government communication, both of which negatively impact government trust. Studies have reported how the swift, decisive communication and actions of the government at the onset of the pandemic had garnered trust in the country.^42^ Other reports have also shown how government trust was damaged by seemingly conflicting government information and reports of government corruption.^43,44^ There is, therefore, a need for government to act decisively when dealing with corruption as this may enhance government trust.^45^

### Implications and recommendations

This study has contributed to the literature highlighting the complexity of issues such as adherence to government measures and vaccine uptake in relation to a pandemic and has identified the role of socioeconomic status in these issues. The implications of this are that future pandemic preparedness cannot be a ‘one-size-fits-all’ approach in a country as diverse as South Africa, particularly given the vast inequities that persist between income groups. It is therefore imperative that pandemic responses do not further widen these inequities. Findings relating to government trust emphasise that this should also be a key consideration in pandemic preparedness and response.

While COVID-19 presented a somewhat unique opportunity to study these issues in the context of a global pandemic, these learnings apply to other public health measures that require the support and cooperation of individuals and communities. These too should be contextually relevant and equity-promoting, drawing on a nuanced understanding of the complexity of human behaviour.

### Strengths and limitations

The use of online survey methodologies has specific advantages during a pandemic when there is a need to collect information quickly, and when there may be challenges to the safe conduct of in-person community surveys. Our Internet-based sampling strategy and survey dissemination via an online panel to a large number of respondents enabled the rapid deployment of a survey to track responses in near real-time, allowing us to study risk perception and behavioural changes during the pandemic. However, a limitation of this approach is the trade-off in terms of population representativeness. As access to devices is limited, we were unable to recruit individuals from the lowest income groups for whom this access would be lowest.

## Conclusion

Results from this study should be used to inform future pandemic preparedness plans. In particular, policymakers should consider the importance of providing scientific information through channels accessible to each socioeconomic group to promote positive behavioural changes, as well as the need to adapt pandemic responses to different socioeconomic groups. As adherence to protective behaviours is linked to government trust, there is a need for government to deal with corruption decisively as this will ensure that it remains a trusted entity.
